# A robust measure of correlation between two genes on a microarray

**DOI:** 10.1186/1471-2105-8-220

**Published:** 2007-06-25

**Authors:** Johanna Hardin, Aya Mitani, Leanne Hicks, Brian VanKoten

**Affiliations:** 1 Department of Mathematics, Pomona College, Claremont, CA 91711, USA; 2 Department of Mathematics, Pitzer College, Claremont, CA 91711, USA; 3 Department of Statistics, University of Nebraska, Lincoln, NE 68588, USA; 4 Department of Mathematics, Lewis and Clark College, Portland, OR 97219, USA

## Abstract

**Background:**

The underlying goal of microarray experiments is to identify gene expression patterns across different experimental conditions. Genes that are contained in a particular pathway or that respond similarly to experimental conditions could be co-expressed and show similar patterns of expression on a microarray. Using any of a variety of clustering methods or gene network analyses we can partition genes of interest into groups, clusters, or modules based on measures of similarity. Typically, Pearson correlation is used to measure distance (or similarity) before implementing a clustering algorithm. Pearson correlation is quite susceptible to outliers, however, an unfortunate characteristic when dealing with microarray data (well known to be typically quite noisy.)

**Results:**

We propose a resistant similarity metric based on Tukey's biweight estimate of multivariate scale and location. The resistant metric is simply the correlation obtained from a resistant covariance matrix of scale. We give results which demonstrate that our correlation metric is much more resistant than the Pearson correlation while being more efficient than other nonparametric measures of correlation (e.g., Spearman correlation.) Additionally, our method gives a systematic gene flagging procedure which is useful when dealing with large amounts of noisy data.

**Conclusion:**

When dealing with microarray data, which are known to be quite noisy, robust methods should be used. Specifically, robust distances, including the biweight correlation, should be used in clustering and gene network analysis.

## 1 Background

One of the primary goals of experiments involving DNA microarrays is to find genes which are somehow similar across various experimental conditions. "Similar" is usually taken to mean co-expressed, but it can be measured in several different ways. The distance (usually one minus similarity) measure most commonly used is Pearson correlation, though Euclidean distance, cosine-angle metric, Spearman rank correlation, and jackknife correlation are also used frequently. (Note that correlation and cosine-angle metrics do not fulfill the triangle inequality, so they are not true distance metrics. However, they are used to measure distance in many applications.) For example, [1-4] use Pearson correlation in their gene network analysis; [5-13] use Pearson correlation (or a modification) to cluster gene expression data. Once the similarity or distance measure is chosen, the relationship between the genes is given by some sort of clustering algorithm (e.g., k-means, hierarchical clustering, *k *nearest neighbors) or gene network analysis.

Clustering results can be highly dependent on the choice of similarity measure (particularly when comparing genes whose similarities are based on tens of samples instead of comparing samples whose similarities are based on thousands of genes); one or two outlying values can produce large changes in the value of some similarity measures. Outlying data points can be real or noise, though microarray data are known to have substantial noise. The noise can occur during any of the stages in the experimental process, and the effect can be in any direction. For example, a large outlier might cause co-expressed genes to seem dissimilar while a different large outlier might cause dissimilar genes to look co-expressed. Sometimes the outlying value is meaningful and important in which case the data should be included in the correlation. Our flagging procedure lets the practitioner determine whether or not a flagged value should be removed.

The goal in our paper is to give a resistant correlation measure that can be used as a distance metric in any clustering or gene network algorithm which calls for some type of distance or similarity measure in order to identify the relationship between a pair of genes, across gene modules, or within a cluster of genes. Tukey's biweight [14] has been well established as a resistant measure of location and scale for multivariate data [15-17]. When considering 2 genes on *n *samples, the 2 × 2 biweight covariance matrix that results from the biweight measurement of multivariate scale can be thought of as a resistant covariance between two genes (or of *n *points in dimension 2). Translating a 2 × 2 biweight covariance matrix into a biweight correlation measure is simply a matter of taking the biweight covariance divided by the product of the individual gene biweight standard deviations (analogous to computing the Pearson correlation from a standard covariance matrix.) Tukey's biweight is a type of M-estimate, a class of estimators which has been used in robust correlation estimates (for example, Mosteller and Tukey defined the cob [18] and Wilcox defined the percentage bend correlation [19].) M-estimates are consistent estimates of multivariate location and shape, so the biweight correlation is estimating the same parameter as the Pearson correlation. We show that our robust correlation based on the biweight M-estimate is intuitive, flexible, and performs well under a variety of data distributions.

When considering the correlation between each pair of genes, we find that, although the biweight correlation and the Pearson correlation usually agree, when they do not agree, there are often problems with the gene's (or genes') data which may indicate to the biologist that the gene should be removed from further study. Our biweight correlation method provides two novel applications particularly suited to microarray analysis: 1. We have created a similarity measure that is resistant to outlying data points (an important feature in analyzing microarray data), and 2. By investigating gene pairs that have discrepant correlation values, we create a diagnostic procedure to identify values which may need to be flagged (i.e., removed or else further investigated.)

In the remainder of the paper, we provide details of the method and results. First, in section 1.1 we discuss microarrays and their particular need for resistant measures. In section 1.2 we explain Tukey's biweight (its computation is given in the appendix, section 8.) We give our results in section 2, showing that the biweight correlation can be used as a resistant similarity measure or a diagnostic procedure for flagging data. We then demonstrate, in section 2.4, that our method is more efficient than Spearman correlation (another resistant correlation method.) In section 2.5 we show that the biweight correlation has empirically low bias and is superior to other robust measures. We conclude with some ideas of how to further develop our methods for other microarray applications.

### 1.1 Why resistance is important in microarray analysis

Microarray technology requires biologists and statisticians to work side by side in analyzing gene expression information. Gene microarray chips measure, simultaneously, the expression levels of thousands of genes in an organism. Comprehensive gene expression data is useful if one wishes to find clusters of genes with similar function. Microarrays have been used to study the gene expression trends (for example across time) in diseases and even to classify and diagnose different types of diseases, such as cancerous tumors [20]. For some organisms, microarrays enable biologists to monitor the entire genome of interest on a single chip in order to create a large picture of the interactions among thousands of genes simultaneously. A microarray is an orderly arrangement of spots that provides a medium for measuring known and unknown DNA pieces (genes) based on base-pairing rules. Each microarray measures thousands of genes simultaneously, so resulting microarray data is typically on the order of thousands of genes by tens of samples.

Although microarray technology has been very useful in discovering changes in gene expression, limitations of the technology have been observed: dye bias and relative gene expression levels having different sample variances due to differences in experimental conditions [21]; differences due to laboratories and platforms [22,23]; pixel saturation [24]; low signal/noise ratio [25]; and differences due to image analysis techniques [26-29]. Researchers have worked to address the particular problems inherent in microarray analyses, but even after novel techniques (of, for example, normalization or filtering) have been applied, microarray data remain noisy [30,31].

Some work has been done showing the need for resistant correlation metrics as similarity measures. In particular, Heyer et al. give a jackknife correlation that is more resistant than the Pearson correlation. However, as they state in their paper, the jackknife correlation is only resistant to single outliers [32].

### 1.2 Biweight as a resistant correlation measure

Tukey's biweight has been used as a resistant estimator of location and scatter as well as a resistant estimator of regression parameters in a wide range of applications (see [33] for an overview of Tukey's work in resistant statistics). The former approach has been used by Affymetrix to normalize microarray data [34] but not in applications of data distances.

M-estimators are a class of estimators of multi-dimensional location and scatter that provide for flexibility, efficiency, and resistance. The key to M-estimation is the ability of the estimator to down-weight points that are far from the data center with respect to the data scatter. Because of the weighting, M-estimates are more resistant to outlying values than standard estimates (like the mean or the Pearson correlation.) Additionally, M-estimates use the actual data values in constructing location and scatter estimates and are therefore more efficient than estimators based on rank (like the median or the Spearman rank correlation.) M-estimators are defined iteratively using a weight function which down-weights data values that are far from the center of the data. We use the M-estimate of 2-dimensional scatter (i.e., covariance) to calculate a biweight correlation. Details for the biweight are given in the appendix, and R code for the biweight is available from the corresponding author (some of the R code is taken from Wilcox [19].)

An important aspect of M-estimators is their resistance to outlying data values. One measure of the resistance of an estimator is its replacement breakdown, which is the smallest fraction of a data set that one could replace with corrupt data in such a way as to take the estimator over all bounds [17]. Unlike the mean (breakdown = 0) or the median (breakdown close to 12
 MathType@MTEF@5@5@+=feaafiart1ev1aaatCvAUfKttLearuWrP9MDH5MBPbIqV92AaeXatLxBI9gBaebbnrfifHhDYfgasaacH8akY=wiFfYdH8Gipec8Eeeu0xXdbba9frFj0=OqFfea0dXdd9vqai=hGuQ8kuc9pgc9s8qqaq=dirpe0xb9q8qiLsFr0=vr0=vr0dc8meaabaqaciaacaGaaeqabaqabeGadaaakeaadaWcaaqaaiabigdaXaqaaiabikdaYaaaaaa@2E9E@), the biweight is parameterized so that the breakdown can be adjusted over a range of values. Adjusting the breakdown value will have implications in flagging data values (discussed further in section 2.3).

The results of the biweight iteration scheme are a multivariate location estimate, T˜
 MathType@MTEF@5@5@+=feaafiart1ev1aaatCvAUfKttLearuWrP9MDH5MBPbIqV92AaeXatLxBI9gBaebbnrfifHhDYfgasaacH8akY=wiFfYdH8Gipec8Eeeu0xXdbba9frFj0=OqFfea0dXdd9vqai=hGuQ8kuc9pgc9s8qqaq=dirpe0xb9q8qiLsFr0=vr0=vr0dc8meaabaqaciaacaGaaeqabaqabeGadaaakeaacuWGubavgaacaaaa@2DEC@, and shape estimate, S˜
 MathType@MTEF@5@5@+=feaafiart1ev1aaatCvAUfKttLearuWrP9MDH5MBPbIqV92AaeXatLxBI9gBaebbnrfifHhDYfgasaacH8akY=wiFfYdH8Gipec8Eeeu0xXdbba9frFj0=OqFfea0dXdd9vqai=hGuQ8kuc9pgc9s8qqaq=dirpe0xb9q8qiLsFr0=vr0=vr0dc8meaabaqaciaacaGaaeqabaqabeGadaaakeaacuWGtbWugaacaaaa@2DEA@. Letting s˜jl
 MathType@MTEF@5@5@+=feaafiart1ev1aaatCvAUfKttLearuWrP9MDH5MBPbIqV92AaeXatLxBI9gBaebbnrfifHhDYfgasaacH8akY=wiFfYdH8Gipec8Eeeu0xXdbba9frFj0=OqFfea0dXdd9vqai=hGuQ8kuc9pgc9s8qqaq=dirpe0xb9q8qiLsFr0=vr0=vr0dc8meaabaqaciaacaGaaeqabaqabeGadaaakeaacuWGZbWCgaacamaaBaaaleaacqWGQbGAcqWGSbaBaeqaaaaa@3114@ be the (*j, l*)^*th *^element of S˜
 MathType@MTEF@5@5@+=feaafiart1ev1aaatCvAUfKttLearuWrP9MDH5MBPbIqV92AaeXatLxBI9gBaebbnrfifHhDYfgasaacH8akY=wiFfYdH8Gipec8Eeeu0xXdbba9frFj0=OqFfea0dXdd9vqai=hGuQ8kuc9pgc9s8qqaq=dirpe0xb9q8qiLsFr0=vr0=vr0dc8meaabaqaciaacaGaaeqabaqabeGadaaakeaacuWGtbWugaacaaaa@2DEA@, we can think of s˜jl
 MathType@MTEF@5@5@+=feaafiart1ev1aaatCvAUfKttLearuWrP9MDH5MBPbIqV92AaeXatLxBI9gBaebbnrfifHhDYfgasaacH8akY=wiFfYdH8Gipec8Eeeu0xXdbba9frFj0=OqFfea0dXdd9vqai=hGuQ8kuc9pgc9s8qqaq=dirpe0xb9q8qiLsFr0=vr0=vr0dc8meaabaqaciaacaGaaeqabaqabeGadaaakeaacuWGZbWCgaacamaaBaaaleaacqWGQbGAcqWGSbaBaeqaaaaa@3114@ as a resistant estimate of cov (**X**_*j*_, **X**_*l*_) (where **X**_*j *_and **X**_*l *_are two *n*-vectors of interest.) Consequently,

r˜jl=s˜jls˜jjs˜ll
 MathType@MTEF@5@5@+=feaafiart1ev1aaatCvAUfKttLearuWrP9MDH5MBPbIqV92AaeXatLxBI9gBaebbnrfifHhDYfgasaacH8akY=wiFfYdH8Gipec8Eeeu0xXdbba9frFj0=OqFfea0dXdd9vqai=hGuQ8kuc9pgc9s8qqaq=dirpe0xb9q8qiLsFr0=vr0=vr0dc8meaabaqaciaacaGaaeqabaqabeGadaaakeaacuWGYbGCgaacamaaBaaaleaacqWGQbGAcqWGSbaBaeqaaOGaeyypa0ZaaSaaaeaacuWGZbWCgaacamaaBaaaleaacqWGQbGAcqWGSbaBaeqaaaGcbaWaaOaaaeaacuWGZbWCgaacamaaBaaaleaacqWGQbGAcqWGQbGAaeqaaOGafm4CamNbaGaadaWgaaWcbaGaemiBaWMaemiBaWgabeaaaeqaaaaaaaa@3F8E@

is the biweight correlation between vectors *j *and *l *and is a more resistant estimate of correlation than the Pearson correlation (denoted by *r*_*jl*_.) Because the components (center and shape parameters) are estimated using resistant techniques (unlike the Pearson correlation), we know the biweight correlation will be more resistant than the Pearson correlation. Note that |r˜jl
 MathType@MTEF@5@5@+=feaafiart1ev1aaatCvAUfKttLearuWrP9MDH5MBPbIqV92AaeXatLxBI9gBaebbnrfifHhDYfgasaacH8akY=wiFfYdH8Gipec8Eeeu0xXdbba9frFj0=OqFfea0dXdd9vqai=hGuQ8kuc9pgc9s8qqaq=dirpe0xb9q8qiLsFr0=vr0=vr0dc8meaabaqaciaacaGaaeqabaqabeGadaaakeaacuWGYbGCgaacamaaBaaaleaacqWGQbGAcqWGSbaBaeqaaaaa@3112@| ≤ 1.

Using the biweight correlation (r˜
 MathType@MTEF@5@5@+=feaafiart1ev1aaatCvAUfKttLearuWrP9MDH5MBPbIqV92AaeXatLxBI9gBaebbnrfifHhDYfgasaacH8akY=wiFfYdH8Gipec8Eeeu0xXdbba9frFj0=OqFfea0dXdd9vqai=hGuQ8kuc9pgc9s8qqaq=dirpe0xb9q8qiLsFr0=vr0=vr0dc8meaabaqaciaacaGaaeqabaqabeGadaaakeaacuWGYbGCgaacaaaa@2E28@) as a resistant estimate of the correlation measure, we can incorporate r˜
 MathType@MTEF@5@5@+=feaafiart1ev1aaatCvAUfKttLearuWrP9MDH5MBPbIqV92AaeXatLxBI9gBaebbnrfifHhDYfgasaacH8akY=wiFfYdH8Gipec8Eeeu0xXdbba9frFj0=OqFfea0dXdd9vqai=hGuQ8kuc9pgc9s8qqaq=dirpe0xb9q8qiLsFr0=vr0=vr0dc8meaabaqaciaacaGaaeqabaqabeGadaaakeaacuWGYbGCgaacaaaa@2E28@ into clustering algorithms which depend on similarities or 1 - r˜
 MathType@MTEF@5@5@+=feaafiart1ev1aaatCvAUfKttLearuWrP9MDH5MBPbIqV92AaeXatLxBI9gBaebbnrfifHhDYfgasaacH8akY=wiFfYdH8Gipec8Eeeu0xXdbba9frFj0=OqFfea0dXdd9vqai=hGuQ8kuc9pgc9s8qqaq=dirpe0xb9q8qiLsFr0=vr0=vr0dc8meaabaqaciaacaGaaeqabaqabeGadaaakeaacuWGYbGCgaacaaaa@2E28@ into clustering algorithms that depend on distances. In the next section we will demonstrate that the biweight correlation is clearly a better choice for a distance (or similarity) measure than the Pearson correlation (*r*).

## 2 Results

Because our methods are most valuable when applied to noisy data, we applied our technique to a real microarray data set. The data set was chosen because it has been used widely in clustering applications [5-7] as well as gene network applications [1-3]. The data are taken from an experiment on *Saccharomyces cerevisiae *created to describe yeast genes with periodically varying transcript levels within the cell cycle [35]. The cell cycle data are based on a time course experiment, and so they are not independent and identically distributed (iid.) However, they are typical of many microarray data sets which are also not iid. The data are publicly available from the Stanford Microarray Database (SMD)  and include 25 samples on over 6000 genes. We kept the default filters from SMD, including using "Log (base2) of R/G Normalized Ratio (Mean)" as our value of interest (that is, we worked with a value that is the log (base2) transformation of the normalized ratio of the average red signal ("R") and the average green signal ("G").) Typically, the red signal measures the amount of gene expression activity under an experimental condition, and the green signal measures the gene expression activity for a control. The value of interest is the relative expression measured by the ratio log_2 _(R/G). The only additional filtering we did was to eliminate genes that had more than ten missing values (correlation was computed on the remaining values for those genes with minor missing data.) Note, also, that we have applied similar techniques to multiple other independent data sets, and the results are consistent across platforms (e.g., oligonucleotide or cDNA), organisms, and normalization techniques (results not shown.)

### 2.1 Biweight correlation as a resistant similarity measure

**Figure 1:**
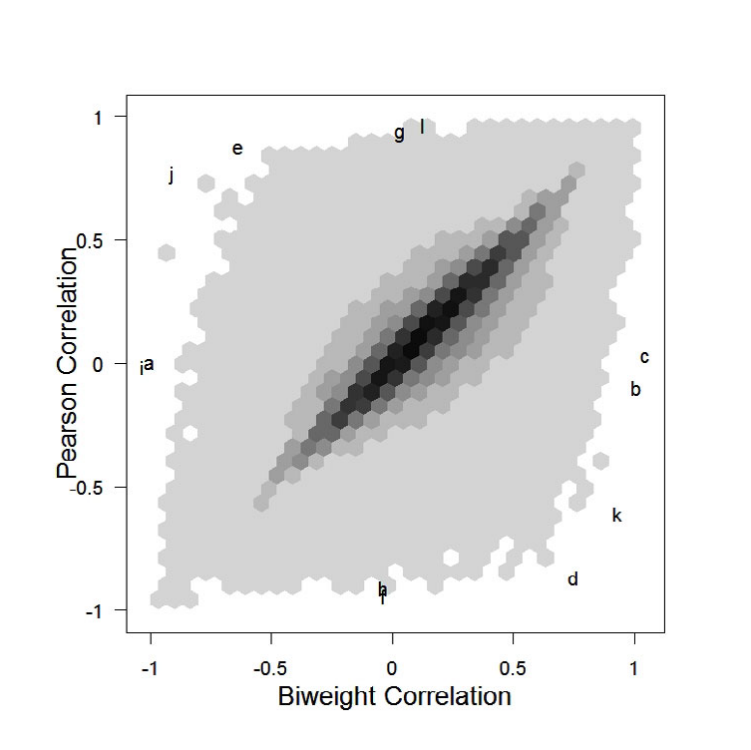
Scatterplot of all pairwise correlations of the 1000 most variable genes in the yeast data. The blackest hexagons represent 9,556 pairs of genes. The lightest hexagons represent one pair of genes. Notice that, though most of the points lie near the line y = x, there are many pairs of genes that give quite different correlations when measured with Pearson's or the biweight. Each letter refers to a gene pair which will be described in figures (2), (3), and (4).

To demonstrate the difference between Pearson correlation (PC) and biweight correlation (BWC), we computed both correlations (BWC based on breakdown of 0.2) on all (10002)
 MathType@MTEF@5@5@+=feaafiart1ev1aaatCvAUfKttLearuWrP9MDH5MBPbIqV92AaeXatLxBI9gBaebbnrfifHhDYfgasaacH8akY=wiFfYdH8Gipec8Eeeu0xXdbba9frFj0=OqFfea0dXdd9vqai=hGuQ8kuc9pgc9s8qqaq=dirpe0xb9q8qiLsFr0=vr0=vr0dc8meaabaqaciaacaGaaeqabaqabeGadaaakeaadaqadaqaauaabeqaceaaaeaacqaIXaqmcqaIWaamcqaIWaamcqaIWaamaeaacqaIYaGmaaaacaGLOaGaayzkaaaaaa@32EE@ pairs of genes from the top 2 most 1000 variable genes (in terms of standard deviation.) A scatterplot with all (10002)
 MathType@MTEF@5@5@+=feaafiart1ev1aaatCvAUfKttLearuWrP9MDH5MBPbIqV92AaeXatLxBI9gBaebbnrfifHhDYfgasaacH8akY=wiFfYdH8Gipec8Eeeu0xXdbba9frFj0=OqFfea0dXdd9vqai=hGuQ8kuc9pgc9s8qqaq=dirpe0xb9q8qiLsFr0=vr0=vr0dc8meaabaqaciaacaGaaeqabaqabeGadaaakeaadaqadaqaauaabeqaceaaaeaacqaIXaqmcqaIWaamcqaIWaamcqaIWaamaeaacqaIYaGmaaaacaGLOaGaayzkaaaaaa@32EE@ pairs of genes is given in figure 1 (the horizontal axis is BWC, the vertical axis is PC.) The PC and BWC are highly positively correlated, with most of the correlations in relative agreement. However, in the corners and on the edges, we see numerous strong discrepancies between the PC and the BWC. A further investigation into those edge points gives clear evidence of why PC and BWC values differ.

Before discussing the particular pairs of interest, we will break down the plot into four (not well defined) groups:

1. gene pairs that give "consistent" PC and BWC

2. gene pairs that give "opposite" PC and BWC

3. gene pairs that give PC ≈ 0 and large |BWC|

4. gene pairs that give large |PC| and BWC ≈ 0

We will discuss group 1 further in section 2.3.

In groups 2–4, the inability to consistently measure gene correlation can generate serious problems in clustering algorithms. We argue that for gene pairs in groups 2–4, the BWC is a much better measure of distance than the PC.

**Figure 2:**
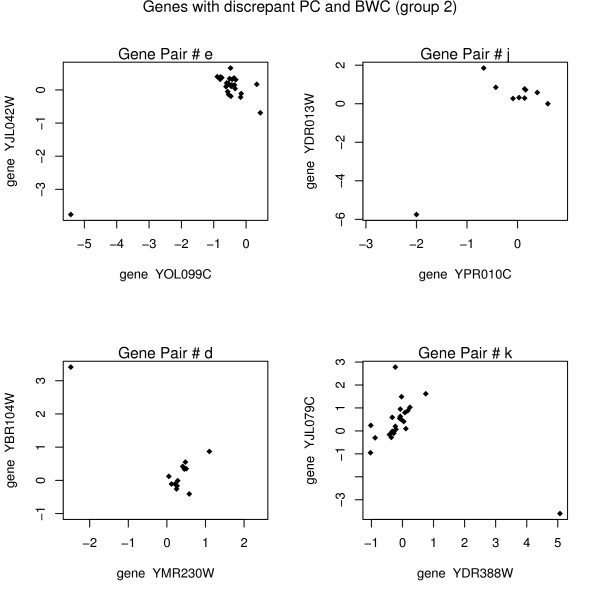
Each point represents the *log*_2 _(*R/G*) value for the specified genes on a particular array. The Pearson correlation and the biweight correlation give opposite values for these group 2 pairs. Due to the outlying values, it is clear that the Pearson correlation is quite misleading.

Consider points e, j, d, and k from figure 1 (group 2 points). For each pair of genes, there is an extreme outlying value causing the PC to be manipulated in the outlier's direction. The panel of plots in figure 2 shows the clear outlying values for each of the points in figure 1 identified as being in group 2.

**Figure 3:**
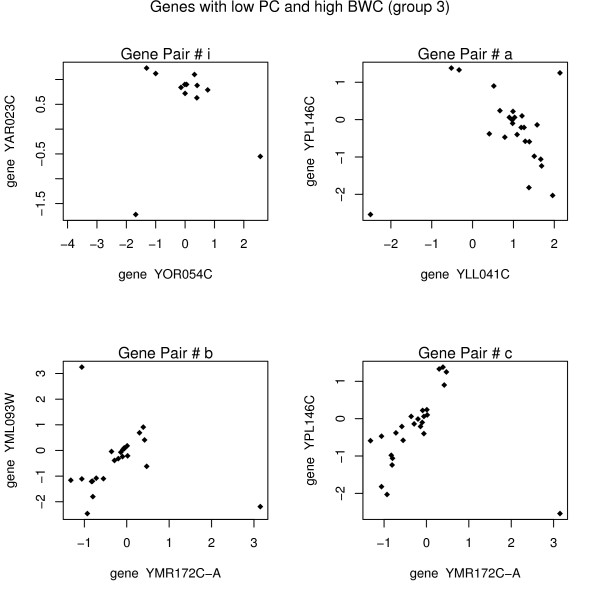
Each point represents the *log*_2 _(*R/G*) value for the specified genes on a particular array. The Pearson correlation is close to zero, and the biweight correlation gives a relatively high value for these group 3 pairs.

Consider points i, a, b, and c from figure 1 (group 3 points). For each pair of genes, there appears to be an outlying point which is nullifying the existing (strong) correlation. The panel of plots in figure 3 shows the existing correlation that has been calculated as low (using PC) because of the outlier(s).

**Figure 4:**
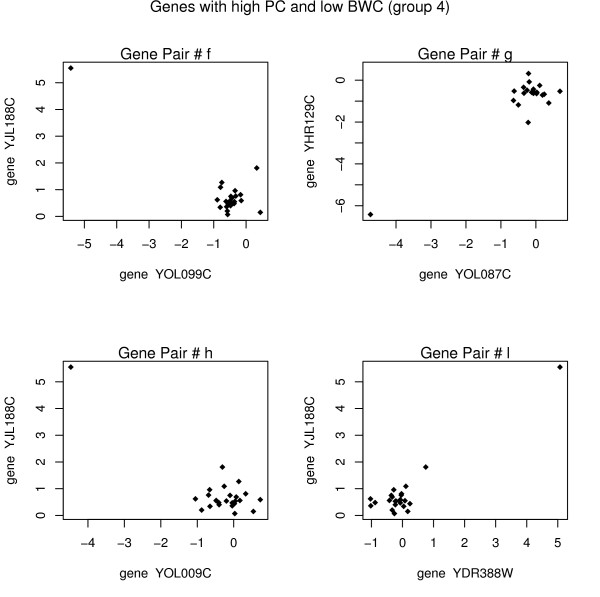
Each point represents the *log*_2 _(*R/G*) value for the specified genes on a particular array. The biweight correlation is close to zero, and the Pearson correlation gives a high absolute correlation. Group 4 pairs are the most worrisome; it would be a mistake to think that two genes were highly correlated when that high correlation is simply due to one outlying point.

Consider points f, g, h, and l from figure 1 (group 4 points). For each pair of genes (seen in figure 4), there appears to be virtually no correlation though the PC calculates a strong correlation. The highly influential points in group 4 are those that we are most worried about. Points in group 4 will show up as strongly co-expressed in either a cluster or a gene network and will give researchers misleading results. Because of the high dimensionality of microarray data, already we often come across false positive results (even without outlying values). Using BWC instead of PC will help to reduce the number of false positives in any given application that are due to outlying values which produce misleadingly high PC.

### 2.2 Using the biweight correlation to flag low quality data

Our method of comparing PC and BWC can also be used as a data flagging method. For gene pairs that produce a relatively high correlation (by at least one of the methods) and highly discrepant correlations across the two methods, we flag the gene pair for further investigation. Note that we require the gene pair to yield a high correlation value (in either direction), because we are not particularly interested in genes whose value flips, for example, from *r *= 0.3 to r˜
 MathType@MTEF@5@5@+=feaafiart1ev1aaatCvAUfKttLearuWrP9MDH5MBPbIqV92AaeXatLxBI9gBaebbnrfifHhDYfgasaacH8akY=wiFfYdH8Gipec8Eeeu0xXdbba9frFj0=OqFfea0dXdd9vqai=hGuQ8kuc9pgc9s8qqaq=dirpe0xb9q8qiLsFr0=vr0=vr0dc8meaabaqaciaacaGaaeqabaqabeGadaaakeaacuWGYbGCgaacaaaa@2E28@ = - 0.2; such a change will not have a strong impact in a clustering scheme because the similarity across the two genes is weak in both measures. We will flag points as outlying if

1. |*r*| > 0.85 OR |r˜
 MathType@MTEF@5@5@+=feaafiart1ev1aaatCvAUfKttLearuWrP9MDH5MBPbIqV92AaeXatLxBI9gBaebbnrfifHhDYfgasaacH8akY=wiFfYdH8Gipec8Eeeu0xXdbba9frFj0=OqFfea0dXdd9vqai=hGuQ8kuc9pgc9s8qqaq=dirpe0xb9q8qiLsFr0=vr0=vr0dc8meaabaqaciaacaGaaeqabaqabeGadaaakeaacuWGYbGCgaacaaaa@2E28@| > 0.85

2. AND |*r *- r˜
 MathType@MTEF@5@5@+=feaafiart1ev1aaatCvAUfKttLearuWrP9MDH5MBPbIqV92AaeXatLxBI9gBaebbnrfifHhDYfgasaacH8akY=wiFfYdH8Gipec8Eeeu0xXdbba9frFj0=OqFfea0dXdd9vqai=hGuQ8kuc9pgc9s8qqaq=dirpe0xb9q8qiLsFr0=vr0=vr0dc8meaabaqaciaacaGaaeqabaqabeGadaaakeaacuWGYbGCgaacaaaa@2E28@| > 1.0

Depending on how strict one wants to be at flagging possible outlying values, one may want to adjust the cutoffs in the above procedure. The quality of data will affect the size of the absolute difference |*r *- r˜
 MathType@MTEF@5@5@+=feaafiart1ev1aaatCvAUfKttLearuWrP9MDH5MBPbIqV92AaeXatLxBI9gBaebbnrfifHhDYfgasaacH8akY=wiFfYdH8Gipec8Eeeu0xXdbba9frFj0=OqFfea0dXdd9vqai=hGuQ8kuc9pgc9s8qqaq=dirpe0xb9q8qiLsFr0=vr0=vr0dc8meaabaqaciaacaGaaeqabaqabeGadaaakeaacuWGYbGCgaacaaaa@2E28@|. Therefore, if a data set has low quality (for example, if GeneSpring output files give evidence of low quality), a resistant metric should be used and/or claims should only be made about genes for which the resistant and non-resistant metrics give similar results.

**Figure 5:**
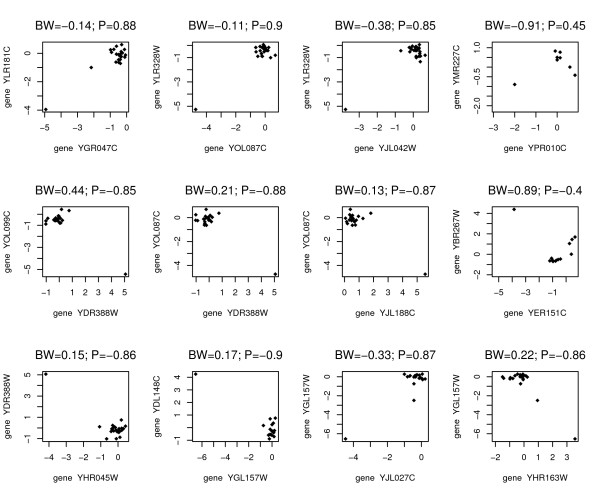
Each point represents the *log*_2 _(*R/G*) value for the specified genes on a particular array. Each panel again shows the relationship between two genes whose biweight and Pearson correlations differ. Here we measure the actual difference between the Pearson correlation and biweight correlation. Each of the 12 pairs of genes had an absolute correlation difference of at least 1.0 and one of the correlations had an absolute value of at least 0.85.

Using the outlying flag procedure defined above, we identified 12 gene pairs (see figure 5.) We can see that in the 12 pairs of genes, genes YOL087C, YDR388W, and YGL157W were identified three times each. Genes which get flagged as outlying when compared to multiple other genes are likely to have some poorly measured data or other outlying virtues. In order to find those genes which are repeatedly seen as outlying, we reduce the flag procedure to 1. a single correlation being at least 0.8 and 2. a difference of at least 0.65. We then identify 593 gene pairs (which represent 363 unique genes) as possibly outlying. By tabulating the frequency of the 363 unique genes in the 593 plots of gene pairs, we are able to find genes which are repeatedly identified (eight genes that showed up in at least 20 of the 593 pairs.) The four most common gene outliers (YLR328W, YDR388W, YJL042W, YJL188C) all showed up in our more stringent outlier detection plot (see figure 5). These gene outliers could represent either noise or truly large values. Follow-up experiments or other datasets would be needed to distinguish these two possibilities.

### 2.3 Effect of breakdown in biweight correlation

As mentioned in section 1.2, the breakdown controls the resistance of the estimator. For example, setting a breakdown at 0.2 allows for up to 20% of the data values to be manipulated without being able to take the estimator across all bounds. Naturally, the lower the breakdown, the less resistant the estimator. In our case, a breakdown of zero will give a BWC estimate almost equivalent to the PC (the very slight difference is due to the BWC weight scheme weighting points differently while PC weights every point 1n
 MathType@MTEF@5@5@+=feaafiart1ev1aaatCvAUfKttLearuWrP9MDH5MBPbIqV92AaeXatLxBI9gBaebbnrfifHhDYfgasaacH8akY=wiFfYdH8Gipec8Eeeu0xXdbba9frFj0=OqFfea0dXdd9vqai=hGuQ8kuc9pgc9s8qqaq=dirpe0xb9q8qiLsFr0=vr0=vr0dc8meaabaqaciaacaGaaeqabaqabeGadaaakeaadaWcaaqaaiabigdaXaqaaiabd6gaUbaaaaa@2F11@.) In a plot (not shown) of the PC vs. BWC at zero breakdown there are no points in the groups we had previously defined as 2, 3, and 4 (see section 2.1.) Conversely, the higher the breakdown, the more discrepant the PC and BWC will be (data not shown.) A higher breakdown will lead to flagging more genes as possibly low quality. Depending on the noise level of the data, one may want to adjust the breakdown. Ideally, the breakdown should be set only as high as the percentage of data which is outlying. A breakdown of 0.2 gives a good balance between resistance and ability to make use of the bulk of the data in the estimation process. The breakdown value will have an effect on the correlation value that is used as a similarity measure. If the breakdown is not high enough, the metric will not be resistant. If the breakdown is too high, the technique will lose power. The effect of the similarity metric on a clustering or gene network will depend on the particular algorithm. However, if the similarities between two genes is estimated to be 0.9 with the first measure and 0.1 with the second measure (or vice versa, both situations seen in figure 1), we would expect clustering algorithms to link the two genes with the first measure and not with the second (or vice versa.) The effect of non-resistant similarity metrics on clustering results can be disastrous.

### 2.4 Efficiency of the biweight correlation

We have demonstrated that the biweight correlation is effective as a resistant correlation as well as a tool for flagging low quality data (both valuable in analyzing microarray data.) The Spearman correlation, based on the ranks of the data, is also a resistant correlation technique. However, because the biweight incorporates the actual data values (instead of just their order), the biweight correlation is more efficient than the Spearman correlation. The efficiency of the biweight as a location and scale estimator has been well studied [17,36]. Table 4 gives the efficiencies (versus the Pearson correlation) for both the biweight and the Spearman correlations. The efficiency is calculated from 10,000 bivariate samples of a given size and correlation. The table values are each the ratio of the variance of the biweight (or Spearman) correlation across the 10,000 samples versus the variance of the Pearson correlation across the 10,000 samples. Particularly for high correlations (those in which we are interested), the biweight correlation is substantially more efficient than the Spearman correlation.

**Figure 6:**
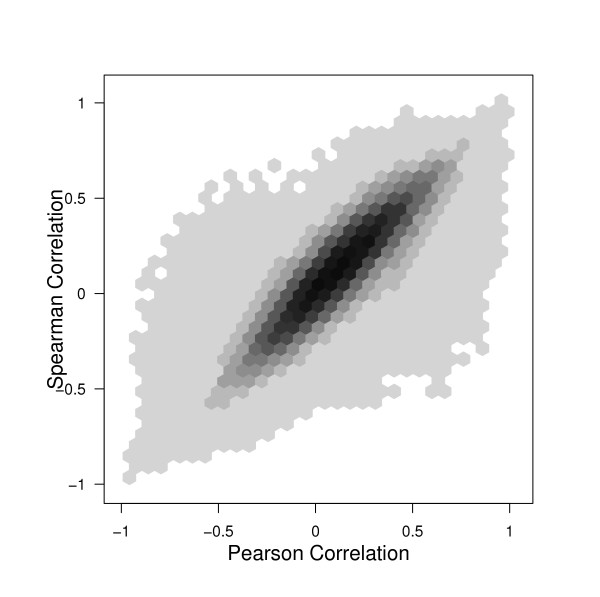
Scatterplot of all pairwise correlations of the 1000 most variable genes in the yeast data. The blackest hexagons represent 7,708 pairs of genes. The lightest hexagons represent one pair of genes. Notice that, unlike the comparison of the biweight correlation vs. Pearson correlation, here there are fewer points in groups 2 or 3 (see section 2.1) indicating that the Spearman correlation is less able to recognize pairs that have mistakenly been given high Pearson (when resistant measure is opposite) or zero Pearson correlations (when resistant measure is high.) Also note that when there is less agreement overall between Spearman and Pearson than between biweight and Pearson (the center section is wider than in figure (1).)

Additionally, in a plot of Spearman vs. Pearson correlations (see figure 6) for the yeast data, we see that the Spearman and Pearson correlations are more consistent with each other than the biweight versus Pearson correlations were (see figure 1) for this noisy data. With clean data we would expect the Pearson and biweight to be more consistent than the Pearson and Spearman. However, because the biweight is able to capture the large correlations that are seen as small with Pearson, the biweight actually seems more different from the Pearson than the Spearman does. The efficiency of the biweight correlation helps us discover large correlations that the Spearman correlation measure misses.

### 2.5 Empirical consistency of biweight correlation under non-normal distributions

In order to assess the performance of the biweight correlation under different situations, we ran a series of simulations computing the empirical correlation for each of different true correlations, sample sizes, distributions, and correlation metrics. For a given true correlation, sample size, and distribution a pair of data were simulated; the empirical correlation was then calculated. In each of the simulations except the one-wild, the correlation structure was imposed after the data were simulated. For the one-wild distribution, the data were simulated with the appropriate correlation structure and then the wild observation was substituted randomly. The process was repeated 10,000 times. The average of the 10,000 simulations (with standard deviations) are seen in tables 1, 2, and 3. Three of the correlation metrics (Pearson, Spearman, and biweight) have already been discussed. The fourth, the percentage bend correlation [19], is an additional robust correlation metric based on M-estimation using Huber's *Ψ*-function. The percentage bend correlation has an advantage over the Spearman in that it uses the weighting of the M-estimation instead of just the ranks of the data, but it has a disadvantage over the biweight because the percentage bend is not iterative and can fail to be as resistant as the biweight.

**Table 1:** 

		Normal	Lognormal	Beta(2,2)	Slash	One-wild
*n *= 15	Pearson	0.487 (0.21)	0.474 (0.19)	0.469 (0.21)	0.298 (0.53)	0.214 (0.26)
	Spearman	0.455 (0.22)	0.627 (0.17)	0.430 (0.23)	0.363 (0.27)	0.393 (0.23)
	Perc. Bend	0.463 (0.22)	0.609 (0.18)	0.429 (0.23)	0.366 (0.29)	0.409 (0.23)
	BWC (brk = 0.1)	0.487 (0.21)	0.511 (0.20)	0.469 (0.22)	0.341 (0.41)	0.317 (0.23)
	BWC (brk = 0.2)	0.485 (0.22)	0.565 (0.21)	0.466 (0.23)	0.401 (0.36)	0.484 (0.22)

*n *= 25	Pearson	0.492 (0.16)	0.484 (0.14)	0.484 (0.16)	0.346 (0.53)	0.265 (0.20)
	Spearman	0.465 (0.17)	0.647 (0.12)	0.447 (0.17)	0.393 (0.21)	0.427 (0.17)
	Perc. Bend	0.468 (0.17)	0.622 (0.12)	0.442 (0.17)	0.393 (0.23)	0.436 (0.17)
	BWC (brk = 0.1)	0.492 (0.16)	0.535 (0.15)	0.483 (0.16)	0.393 (0.33)	0.463 (0.17)
	BWC (brk = 0.2)	0.491 (0.16)	0.562 (0.16)	0.481 (0.17)	0.436 (0.27)	0.490 (0.17)
	BWC (brk = 0.4)	0.483 (0.23)	0.580 (0.20)	0.468 (0.24)	0.477 (0.26)	0.483 (0.22)

*n *= 50	Pearson	0.494 (0.11)	0.494 (0.10)	0.493 (0.11)	0.388 (0.52)	0.334 (0.14)
	Spearman	0.472 (0.11)	0.659 (0.08)	0.459 (0.12)	0.414 (0.15)	0.454 (0.12)
	Perc. Bend	0.471 (0.11)	0.625 (0.09)	0.449 (0.12)	0.416 (0.16)	0.458 (0.12)
	BWC (brk = 0.1)	0.494 (0.11)	0.533 (0.11)	0.493 (0.11)	0.446 (0.23)	0.490 (0.11)
	BWC (brk = 0.2)	0.494 (0.11)	0.544 (0.12)	0.492 (0.12)	0.472 (0.18)	0.495 (0.11)
	BWC (brk = 0.4)	0.491 (0.15)	0.553(0.14)	0.486 (0.16)	0.490 (0.17)	0.492 (0.15)

At each sample size (*n*), correlation metric, and data distribution (*ρ *= 0.5), the average correlation is reported (standard deviation in parentheses) for 10,000 random samples. The correlation metrics compared are Pearson correlation, Spearman correlation, percentage bend correlation, and biweight correlation at three different breakdown values. Distributions compared are normal, lognormal (skewed), beta(2,2) (light tails), slash (heavy tails), and one-wild (outlier). (Note that a breakdown of 0.4 with *n *= 15 data points leaves too few points to accurately estimate a correlation.)

**Table 2:** 

		Normal	Lognormal	Beta(2,2)	Slash	One-wild
*n *= 15	Pearson	0.686 (0.15)	0.677 (0.13)	0.671 (0.16)	0.433 (0.49)	0.297 (0.26)
	Spearman	0.646 (0.17)	0.780 (0.12)	0.622 (0.18)	0.528 (0.24)	0.561 (0.19)
	Perc. Bend	0.660 (0.17)	0.779 (0.12)	0.628 (0.18)	0.530 (0.25)	0.583 (0.19)
	BWC (brk = 0.1)	0.685 (0.17)	0.704 (0.14)	0.670 (0.16)	0.504 (0.36)	0.519 (0.22)
	BWC (brk = 0.2)	0.683 (0.15)	0.749 (0.14)	0.667 (0.16)	0.583 (0.31)	0.685 (0.16)

*n *= 25	Pearson	0.693 (0.11)	0.685 (0.10)	0.686 (0.11)	0.493 (0.48)	0.373 (0.20)
	Spearman	0.662 (0.12)	0.802 (0.08)	0.643 (0.13)	0.571 (0.18)	0.610 (0.14)
	Perc. Bend	0.668 (0.12)	0.789 (0.09)	0.643 (0.13)	0.572 (0.19)	0.624 (0.13)
	BWC (brk = 0.1)	0.693 (0.11)	0.725 (0.10)	0.685 (0.12)	0.581 (0.28)	0.681 (0.12)
	BWC (brk = 0.2)	0.692 (0.12)	0.747 (0.11)	0.684 (0.12)	0.642 (0.22)	0.693 (0.12)
	BWC (brk = 0.4)	0.685 (0.16)	0.762 (0.13)	0.670 (0.18)	0.678 (0.19)	0.688 (0.15)

*n *= 50	Pearson	0.697 (0.07)	0.693 (0.07)	0.693 (0.08)	0.560 (0.46)	0.474 (0.13)
	Spearman	0.672 (0.08)	0.815 (0.05)	0.655 (0.09)	0.601 (0.12)	0.647 (0.09)
	Perc. Bend	0.673 (0.08)	0.793 (0.06)	0.650 (0.09)	0.603 (0.13)	0.653 (0.09)
	BWC (brk = 0.1)	0.697 (0.08)	0.727 (0.08)	0.693 (0.08)	0.640 (0.18)	0.696 (0.08)
	BWC (brk = 0.2)	0.697 (0.08)	0.738 (0.08)	0.692 (0.08)	0.675 (0.14)	0.698 (0.08)
	BWC (brk = 0.4)	0.694 (0.10)	0.745 (0.10)	0.686 (0.11)	0.692 (0.12)	0.695 (0.10)

At each sample size (*n*), correlation metric, and data distribution (*ρ *= 0.7), the average correlation is reported (standard deviation in parentheses) for 10,000 random samples. The correlation metrics compared are Pearson correlation, Spearman correlation, percentage bend correlation, and biweight correlation at three different breakdown values. Distributions compared are normal, lognormal (skewed), beta(2,2) (light tails), slash (heavy tails), and one-wild (outlier). (Note that a breakdown of 0.4 with *n *= 15 data points leaves too few points to accurately estimate a correlation.)

**Table 3:** 

		Normal	Lognormal	Beta(2,2)	Slash	One-wild
*n *= 15	Pearson	0.894 (0.06)	0.887 (0.05)	0.885 (0.07)	0.588 (0.43)	0.389 (0.26)
	Spearman	0.860 (0.08)	0.917 (0.05)	0.841 (0.09)	0.723 (0.18)	0.745 (0.14)
	Perc. Bend	0.879 (0.07)	0.927 (0.05)	0.859 (0.09)	0.722 (0.19)	0.772 (0.14)
	BWC (brk = 0.1)	0.894 (0.06)	0.898 (0.05)	0.885 (0.07)	0.721 (0.29)	0.860 (0.11)
	BWC (brk = 0.2)	0.893 (0.06)	0.918 (0.05)	0.883 (0.07)	0.817 (0.21)	0.892 (0.06)

*n *= 25	Pearson	0.897 (0.04)	0.894 (0.04)	0.892 (0.04)	0.671 (0.39)	0.481 (0.20)
	Spearman	0.871 (0.06)	0.931 (0.03)	0.858 (0.06)	0.774 (0.13)	0.802 (0.09)
	Perc. Bend	0.882 (0.05)	0.931 (0.03)	0.868 (0.06)	0.774 (0.13)	0.820 (0.08)
	BWC (brk = 0.1)	0.897 (0.04)	0.910 (0.04)	0.891 (0.05)	0.809 (0.18)	0.896 (0.04)
	BWC (brk = 0.2)	0.897 (0.05)	0.920 (0.04)	0.890 (0.05)	0.870 (0.11)	0.896 (0.05)
	BWC (brk = 0.4)	0.893 (0.06)	0.925 (0.05)	0.882 (0.08)	0.889 (0.08)	0.893 (0.06)

*n *= 50	Pearson	0.899 (0.03)	0.897 (0.03)	0.896 (0.03)	0.757 (0.34)	0.606 (0.13)
	Spearman	0.882 (0.04)	0.939 (0.02)	0.870 (0.04)	0.811 (0.08)	0.845 (0.05)
	Perc. Bend	0.886 (0.03)	0.933 (0.02)	0.874 (0.04)	0.811 (0.09)	0.854 (0.05)
	BWC (brk = 0.1)	0.899 (0.03)	0.911 (0.03)	0.896 (0.03)	0.863 (0.09)	0.898 (0.03)
	BWC (brk = 0.2)	0.899 (0.03)	0.916 (0.03)	0.895 (0.03)	0.890 (0.06)	0.898 (0.03)
	BWC (brk = 0.4)	0.898 (0.04)	0.918 (0.04)	0.892 (0.04)	0.895 (0.05)	0.896 (0.04)

At each sample size (*n*), correlation metric, and data distribution (*ρ *= 0.9), the average correlation is reported (standard deviation in parentheses) for 10,000 random samples. The correlation metrics compared are Pearson correlation, Spearman correlation, percentage bend correlation, and biweight correlation at three different breakdown values. Distributions compared are normal, lognormal (skewed), beta(2,2) (light tails), slash (heavy tails), and one-wild (outlier). (Note that a breakdown of 0.4 with *n *= 15 data points leaves too few points to accurately estimate a correlation.)

**Table 4:** 

		true correlation					
		0.5		0.7		0.9	
		BWC	SP	BWC	SP	BWC	SP
sample size	10	0.894	0.918	0.874	0.802	0.853	0.529
	20	0.900	0.894	0.896	0.788	0.892	0.535
	50	0.898	0.878	0.915	0.795	0.920	0.607
	100	0.910	0.888	0.912	0.791	0.908	0.608

Efficiency of biweight correlation (BWC) vs. Spearman (SP) correlation. All values are simulated variance of correlation of interest (biweight or Spearman) divided by the variance of the Pearson correlation. Simulations are based on 10,000 samples of standard normal bivariate data sets with the appropriate sample size and correlation.

The distributions of data are meant to cover a variety of situations. The Lognormal data are skewed; the Beta(2,2) data have light tails. The slash distribution is created by dividing a standard normal deviate by an independent uniform (0,1) deviate and has much heavier tails than normal while being less pathological than the Cauchy distribution. The one-wild distribution is a contaminated standard normal such that one value (in only one dimension) is replaced with a random deviate from a uniform (5,10) distribution. From tables 1, 2, and 3 we make the following observations:

• Pearson correlation is seriously affected by heavy tails (slash distribution) and outliers (one-wild distribution.)

• Spearman and percentage bend correlations are quite resistant, but they tend to underperform the biweight (at any breakdown).

• The biweight correlation performs well consistently across different distributions and sample sizes.

• Though some efficiency is lost when the biweight is compared to the Pearson, the improvement in performance for non-normal data is essential for applications to microarray data.

• The breakdown parameter alters the biweight correlation performance only slightly. As long as the breakdown percentage covers the amount of the contamination, the biweight correlation will have low bias and high efficiency.

### 2.6 Convergence of the biweight

As described in section 1.2, the biweight correlation is an iterative estimator. For a (normal) sample size of 25, it takes about 43 seconds to compute 100,000 pairwise biweight correlations on a Pentium 4, 3GHz computer with 2GB RAM running Windows XP (compared to less than a second for the Pearson correlation and the Spearman correlation, and about 2 seconds for the percentage bend correlation.) Admittedly, the computation time is the shortcoming for the biweight correlation when computing all pairwise correlations across hundreds or thousands of genes. All simulations are done using R and would likely be considerably faster using a different programming language.

As mentioned in the appendix, the biweight correlation is computed by first finding an initial estimate of location and scatter. We have found that initializing the biweight using robust estimates of location and scatter of the median and MAD (Median of the Absolute Deviations from the median) converge to the same biweight estimates as using the Minimum Covariance Determinant [37] location and scatter estimates (which are slightly better multivariate estimates but slower to compute.) Additionally, we have found (simulations not shown) that running the iteration for 5–10 steps gives equivalent results (biweight correlations). Typically, for noisy data, the iteration scheme will take between 10–25 steps to converge fully. For a slash distribution with a sample of size 25, it takes 95 seconds to compute 100,000 pairwise biweight correlations which completely converge and 42 seconds when the iterations are capped at 8.

## 3 Discussion

We have provided a novel resistant estimate of correlation based on a well-known multivariate location estimator of location and scale. Tukey's biweight has been used as a resistant estimator in diverse contexts such as regression, analysis of variance, time series, and control charts to monitor product quality [33] because of its resistance and efficiency properties. We have shown that the biweight is also a powerful technique to use when computing correlations between pairs of genes regardless of whether there is a significant amount of contamination or not.

Additionally, the tuning parameters of the biweight allow for the estimates to be minimally or largely resistant to outlying values; the breakdown of the correlation (which defines the tuning parameters) can be set to allow for a degree of resistance suitable for the analysis. We have found that setting our breakdown to 0.2 works well in most situations.

Not only does the biweight correlation give a resistant measure of correlation, but it also provides a data flagging method that (a) finds pairs of genes which give misleading Pearson correlations, and (b) finds genes that, when compared to many other genes, consistently give misleading Pearson correlations. The data flagging method can be used to improve the accuracy of secondary analyses (e.g., clustering or gene network analyses) and to decrease the rate of false positives. The high dimensionality of microarray data produces a need for automated data cleaning, and we provide one way of examining the data for outlying values before analyses are performed.

Because the biweight estimator is iterative, it is computationally more time intensive than either the Pearson or the Spearman correlation estimates. To save computation time, one might use the biweight as an initial estimate and an outlier detection method, and then progress to one of the other methods for analyses that require computing correlations multiple times in a row.

We have used microarray data to illustrate our methods. However, the methods can easily be applied to any data set, and they will be particularly useful for data sets where there is a large amount of noise and many distance pairs are being calculated. For example, we could use this method on other high throughput data like proteomic or metabolomic data. Additionally, other disciplines with large data sets like Astronomy and Econometrics will also value a robust and systematic procedure for calculating distances. Many supervised discrimination techniques use metrics/statistics which are similar to correlations. For example, Fisher's Linear Discriminant Analysis (LDA) is based on the mahalanobis squared distance. Because the biweight is inherently a multivariate estimator, one could easily use the biweight to measure resistant mahalanobis squared distances to use in LDA. Additionally, popular methods like Classification and Regression Trees (CART) use regression models to partition samples into groups. Biweight regression methods could also be used to make for more resistant partitioning in CART methods.

## 4 Conclusion

Tukey's biweight has been well established as a resistant estimation method in many fields. It has played a small role in the analyses of microarray data. However, the need for resistant methods in microarray data is great, and the biweight is a powerful tool that can provide improved methodology and results in many applications of microarray analyses. The methods shown here use Tukey's biweight to give a robust and efficient estimate of distance between two genes on a microarray.

## 5 Availability

R code is available from the authors as well as in a supplementary file to this article.

## 6 Authors' contributions

JH conceived of the study, participated in its design and coordination, and wrote the manuscript. AM, LH, and BVK contributed to writing the computer code, validating the method, and editing the manuscript.

## 7 Appendix

Multivariate estimates of location and scatter given by constrained M-estimates are defined iteratively using a weight function, *w *(·), to down-weight data values that are far from the bulk of the data. Using initial estimates of the location, T˜
 MathType@MTEF@5@5@+=feaafiart1ev1aaatCvAUfKttLearuWrP9MDH5MBPbIqV92AaeXatLxBI9gBaebbnrfifHhDYfgasaacH8akY=wiFfYdH8Gipec8Eeeu0xXdbba9frFj0=OqFfea0dXdd9vqai=hGuQ8kuc9pgc9s8qqaq=dirpe0xb9q8qiLsFr0=vr0=vr0dc8meaabaqaciaacaGaaeqabaqabeGadaaakeaacuWGubavgaacaaaa@2DEC@, and shape, S˜
 MathType@MTEF@5@5@+=feaafiart1ev1aaatCvAUfKttLearuWrP9MDH5MBPbIqV92AaeXatLxBI9gBaebbnrfifHhDYfgasaacH8akY=wiFfYdH8Gipec8Eeeu0xXdbba9frFj0=OqFfea0dXdd9vqai=hGuQ8kuc9pgc9s8qqaq=dirpe0xb9q8qiLsFr0=vr0=vr0dc8meaabaqaciaacaGaaeqabaqabeGadaaakeaacuWGtbWugaacaaaa@2DEA@, we calculate the distance of each point to the center of the data set,

dj=(Xj−T˜)′S˜−1(Xj−T˜)
 MathType@MTEF@5@5@+=feaafiart1ev1aaatCvAUfKttLearuWrP9MDH5MBPbIqV92AaeXatLxBI9gBaebbnrfifHhDYfgasaacH8akY=wiFfYdH8Gipec8Eeeu0xXdbba9frFj0=OqFfea0dXdd9vqai=hGuQ8kuc9pgc9s8qqaq=dirpe0xb9q8qiLsFr0=vr0=vr0dc8meaabaqaciaacaGaaeqabaqabeGadaaakeaacqWGKbazdaWgaaWcbaGaemOAaOgabeaakiabg2da9maakaaabaGaeiikaGccbeGae8hwaG1aaSbaaSqaaiabdQgaQbqabaGccqGHsislcuWGubavgaacaiqbcMcaPyaafaGafm4uamLbaGaadaahaaWcbeqaaiabgkHiTiabigdaXaaakiabcIcaOiab=HfaynaaBaaaleaacqWGQbGAaeqaaOGaeyOeI0IafmivaqLbaGaacqGGPaqkaSqabaaaaa@4167@

For a given objective function, *ρ *(·), the constraint, parameterized by *k*, (for the constrained M-estimator) is (see [38] for details)

n−1∑j=1nρ(djk)=bo
 MathType@MTEF@5@5@+=feaafiart1ev1aaatCvAUfKttLearuWrP9MDH5MBPbIqV92AaeXatLxBI9gBaebbnrfifHhDYfgasaacH8akY=wiFfYdH8Gipec8Eeeu0xXdbba9frFj0=OqFfea0dXdd9vqai=hGuQ8kuc9pgc9s8qqaq=dirpe0xb9q8qiLsFr0=vr0=vr0dc8meaabaqaciaacaGaaeqabaqabeGadaaakeaacqWGUbGBdaahaaWcbeqaaiabgkHiTiabigdaXaaakmaaqahabaacciGae8xWdi3aaeWaaeaadaWcaaqaaiabdsgaKnaaBaaaleaacqWGQbGAaeqaaaGcbaGaem4AaSgaaaGaayjkaiaawMcaaaWcbaGaemOAaOMaeyypa0JaeGymaedabaGaemOBa4ganiabggHiLdGccqGH9aqpcqWGIbGydaWgaaWcbaGaem4Ba8gabeaaaaa@42B2@

where *b*_*o *_= *E *[*ρ *(*d/k*)] and *n *is the number of samples (*d *is defined originally as above in equation (2) and subsequently as below in equation (6), and *k *is found using equation (3) after *b*_*o *_and *d*_*j *_are determined.) *b*_*o *_is given as the product of the specified breakdown and the maximum value of *ρ *[39]. To find *k*, the expected value (*b*_*o*_) is calculated under the assumption of multivariate normality (*d *will have a chi-square distribution if the data are normally distributed). Though we retain the convention, we do not presume to think that microarray data are normally distributed. The implication of an incorrect normality assumption will be a breakdown value slightly different from what we set. Because our work does not focus on a particular breakdown value of interest (and instead focuses on the general idea of having a resistant estimation procedure), we are not bothered by a slight miscalculation of the breakdown value.

Subject to the constraint in equation (3), the M-estimators of location and scale are given by the following iterative equations [40,41]

T˜(i+1)=∑jw(dj(i)/k(i))Xj∑jw(dj(i)/k(i))
 MathType@MTEF@5@5@+=feaafiart1ev1aaatCvAUfKttLearuWrP9MDH5MBPbIqV92AaeXatLxBI9gBaebbnrfifHhDYfgasaacH8akY=wiFfYdH8Gipec8Eeeu0xXdbba9frFj0=OqFfea0dXdd9vqai=hGuQ8kuc9pgc9s8qqaq=dirpe0xb9q8qiLsFr0=vr0=vr0dc8meaabaqaciaacaGaaeqabaqabeGadaaakeaacuWGubavgaacamaaCaaaleqabaGaeiikaGIaemyAaKMaey4kaSIaeGymaeJaeiykaKcaaOGaeyypa0ZaaSaaaeaadaaeqaqaaiabdEha3jabcIcaOiabdsgaKnaaDaaaleaacqWGQbGAaeaacqGGOaakcqWGPbqAcqGGPaqkaaGccqGGVaWlcqWGRbWAdaahaaWcbeqaaiabcIcaOiabdMgaPjabcMcaPaaakiabcMcaPGqabiab=HfaynaaBaaaleaacqWGQbGAaeqaaaqaaiabdQgaQbqab0GaeyyeIuoaaOqaamaaqababaGaem4DaCNaeiikaGIaemizaq2aa0baaSqaaiabdQgaQbqaaiabcIcaOiabdMgaPjabcMcaPaaakiabc+caViabdUgaRnaaCaaaleqabaGaeiikaGIaemyAaKMaeiykaKcaaOGaeiykaKcaleaacqWGQbGAaeqaniabggHiLdaaaaaa@5AA7@

S˜(i+1)=∑jw(dj(i)/k(i))(Xj−T˜(i+1))(Xj−T˜(i+1))′∑jv(dj(i)/k(i))
 MathType@MTEF@5@5@+=feaafiart1ev1aaatCvAUfKttLearuWrP9MDH5MBPbIqV92AaeXatLxBI9gBaebbnrfifHhDYfgasaacH8akY=wiFfYdH8Gipec8Eeeu0xXdbba9frFj0=OqFfea0dXdd9vqai=hGuQ8kuc9pgc9s8qqaq=dirpe0xb9q8qiLsFr0=vr0=vr0dc8meaabaqaciaacaGaaeqabaqabeGadaaakeaacuWGtbWugaacamaaCaaaleqabaGaeiikaGIaemyAaKMaey4kaSIaeGymaeJaeiykaKcaaOGaeyypa0ZaaSaaaeaadaaeqaqaaiabdEha3jabcIcaOiabdsgaKnaaDaaaleaacqWGQbGAaeaacqGGOaakcqWGPbqAcqGGPaqkaaGccqGGVaWlcqWGRbWAdaahaaWcbeqaaiabcIcaOiabdMgaPjabcMcaPaaakiabcMcaPiabcIcaOGqabiab=HfaynaaBaaaleaacqWGQbGAaeqaaOGaeyOeI0IafmivaqLbaGaadaahaaWcbeqaaiabcIcaOiabdMgaPjabgUcaRiabigdaXiabcMcaPaaakiabcMcaPiabcIcaOiab=HfaynaaBaaaleaacqWGQbGAaeqaaOGaeyOeI0IafmivaqLbaGaadaahaaWcbeqaaiabcIcaOiabdMgaPjabgUcaRiabigdaXiabcMcaPaaakiqbcMcaPyaafaaaleaacqWGQbGAaeqaniabggHiLdaakeaadaaeqaqaaiabdAha2jabcIcaOiabdsgaKnaaDaaaleaacqWGQbGAaeaacqGGOaakcqWGPbqAcqGGPaqkaaGccqGGVaWlcqWGRbWAdaahaaWcbeqaaiabcIcaOiabdMgaPjabcMcaPaaakiabcMcaPaWcbaGaemOAaOgabeqdcqGHris5aaaaaaa@6F76@

dj(i+1)=(Xj−T˜(i+1))′(S˜(i+1))−1(Xj−T˜(i+1))
 MathType@MTEF@5@5@+=feaafiart1ev1aaatCvAUfKttLearuWrP9MDH5MBPbIqV92AaeXatLxBI9gBaebbnrfifHhDYfgasaacH8akY=wiFfYdH8Gipec8Eeeu0xXdbba9frFj0=OqFfea0dXdd9vqai=hGuQ8kuc9pgc9s8qqaq=dirpe0xb9q8qiLsFr0=vr0=vr0dc8meaabaqaciaacaGaaeqabaqabeGadaaakeaacqWGKbazdaqhaaWcbaGaemOAaOgabaGaeiikaGIaemyAaKMaey4kaSIaeGymaeJaeiykaKcaaOGaeyypa0ZaaOaaaeaacqGGOaakieqacqWFybawdaWgaaWcbaGaemOAaOgabeaakiabgkHiTiqbdsfauzaaiaWaaWbaaSqabeaacqGGOaakcqWGPbqAcqGHRaWkcqaIXaqmcqGGPaqkaaGccuGGPaqkgaqbaiabcIcaOiqbdofatzaaiaWaaWbaaSqabeaacqGGOaakcqWGPbqAcqGHRaWkcqaIXaqmcqGGPaqkaaGccqGGPaqkdaahaaWcbeqaaiabgkHiTiabigdaXaaakiabcIcaOiab=HfaynaaBaaaleaacqWGQbGAaeqaaOGaeyOeI0IafmivaqLbaGaadaahaaWcbeqaaiabcIcaOiabdMgaPjabgUcaRiabigdaXiabcMcaPaaakiabcMcaPaWcbeaaaaa@573B@

(alternating with equation (3) to determine *k*, note that the value for *k *changes at each iteration) where **X**_*j *_is a gene of interest and

ψ(d)=∂ρ(d)∂d
 MathType@MTEF@5@5@+=feaafiart1ev1aaatCvAUfKttLearuWrP9MDH5MBPbIqV92AaeXatLxBI9gBaebbnrfifHhDYfgasaacH8akY=wiFfYdH8Gipec8Eeeu0xXdbba9frFj0=OqFfea0dXdd9vqai=hGuQ8kuc9pgc9s8qqaq=dirpe0xb9q8qiLsFr0=vr0=vr0dc8meaabaqaciaacaGaaeqabaqabeGadaaakeaaiiGacqWFipqEcqGGOaakcqWGKbazcqGGPaqkcqGH9aqpdaWcaaqaaiabgkGi2kab=f8aYjabcIcaOiabdsgaKjabcMcaPaqaaiabgkGi2kabdsgaKbaaaaa@3B75@

w(d)=ψ(d)d
 MathType@MTEF@5@5@+=feaafiart1ev1aaatCvAUfKttLearuWrP9MDH5MBPbIqV92AaeXatLxBI9gBaebbnrfifHhDYfgasaacH8akY=wiFfYdH8Gipec8Eeeu0xXdbba9frFj0=OqFfea0dXdd9vqai=hGuQ8kuc9pgc9s8qqaq=dirpe0xb9q8qiLsFr0=vr0=vr0dc8meaabaqaciaacaGaaeqabaqabeGadaaakeaacqWG3bWDcqGGOaakcqWGKbazcqGGPaqkcqGH9aqpdaWcaaqaaGGaciab=H8a5jabcIcaOiabdsgaKjabcMcaPaqaaiabdsgaKbaaaaa@3865@

*v*(*d*) = *d ψ*(*d*)

The objective function for Tukey's biweight [42] is given by:

ρ(di)={c26[1−(1−(dic)2)3]|di|≤c16|di|>c
 MathType@MTEF@5@5@+=feaafiart1ev1aaatCvAUfKttLearuWrP9MDH5MBPbIqV92AaeXatLxBI9gBaebbnrfifHhDYfgasaacH8akY=wiFfYdH8Gipec8Eeeu0xXdbba9frFj0=OqFfea0dXdd9vqai=hGuQ8kuc9pgc9s8qqaq=dirpe0xb9q8qiLsFr0=vr0=vr0dc8meaabaqaciaacaGaaeqabaqabeGadaaakeaaiiGacqWFbpGCcqGGOaakcqWGKbazdaWgaaWcbaGaemyAaKgabeaakiabcMcaPiabg2da9maaceqabaqbaeaabiGaaaqaamaalaaabaGaem4yam2aaWbaaSqabeaacqaIYaGmaaaakeaacqaI2aGnaaGaei4waSLaeGymaeJaeyOeI0IaeiikaGIaeGymaeJaeyOeI0YaaeWaaeaadaWcaaqaaiabdsgaKnaaBaaaleaacqWGPbqAaeqaaaGcbaGaem4yamgaaaGaayjkaiaawMcaamaaCaaaleqabaGaeGOmaidaaOGaeiykaKYaaWbaaSqabeaacqaIZaWmaaGccqGGDbqxaeaadaabdaqaaiabdsgaKnaaBaaaleaacqWGPbqAaeqaaaGccaGLhWUaayjcSdGaeyizImQaem4yamgabaWaaSaaaeaacqaIXaqmaeaacqaI2aGnaaaabaWaaqWaaeaacqWGKbazdaWgaaWcbaGaemyAaKgabeaaaOGaay5bSlaawIa7aiabg6da+iabdogaJbaaaiaawUhaaaaa@5C20@

Because *E *[*ρ *(·)] is a function of *c *and the breakdown, we can use the Newton-Raphson method to find *c *using *E *[*ρ *(·)] and the breakdown. For example, given a sample in dimension two (*p *= 2), with a breakdown of 0.2, *c *will be 5.07.

The iterative scheme has the potential for the existence of multiple solutions, although multiple solutions essentially never happen in practice. After our iterative scheme has converged, we are left with a location vector and shape matrix. As seen in equation (1), the biweight correlation will be the biweight covariance divided by the product of the individual gene biweight standard deviations.

## Supplementary Material

Additional file 1 R-code for translated biweight. Annotated R code for computing the translated biweight. Includes both code and a worked example. Click here for file
